# Supplementary material for: Complexome profiling of the Chlamydomonas psb28 mutant reveals TEF5 as an early PSII assembly factor
Source: Plant Cell. 2025 Mar 17;37(6):koaf055. doi: 10.1093/plcell/koaf055 (PMC12164590; doi:10.1093/plcell/koaf055)
Supplement: koaf055_Supplementary_Data [file koaf055_supplementary_data.zip › psb28_interactive.zip!/plots/intensity/qualityPlots/peptides/_Cre10.g428550.t1.2.html]

### Cre10.g428550.t1.2

Trivial name:   
SeqCoverage [median]: 4.10  
Mass: 66.36 kDa  
  
 

| Sequence | MissedCleavage | Mass [Da] | wtIntensity | muIntensity | Score |
| --- | --- | --- | --- | --- | --- |
| LTPEEELAR | 0 | 1056.55 | 0.00 | 412.83 | 52.48 |
| TAEGNVQLLR | 0 | 1099.60 | 843.90 | 1486.28 | 204.34 |
| VADAVYRG | 1 | 849.43 | 0.00 | 1267.42 | 135.87 |
|  |  |  | 843.9 | 3166.5 |  |
